# Bactericidal Synergism between Phage YC#06 and Antibiotics: a Combination Strategy to Target Multidrug-Resistant Acinetobacter baumannii *In Vitro* and *In Vivo*

**DOI:** 10.1128/spectrum.00096-22

**Published:** 2022-06-23

**Authors:** Jun Luo, Libo Xie, Min Liu, Qianyuan Li, Peng Wang, Chunhua Luo

**Affiliations:** a The First College of Clinical Medical Science, China Three Gorges University, Yichang, Hubei, China; b Yichang Central People's Hospital, Yichang, Hubei, China; c Central Laboratory, The First College of Clinical Medical Science, China Three Gorges University & Yichang Central People's Hospital, Yichang, Hubei, China; University of Manitoba

**Keywords:** phage-antibiotic combinations, *in vitro*, *in vivo*, multidrug-resistant *A. baumannii*, phage-antibiotic synergy

## Abstract

Phage-antibiotic combination (PAC) therapy is a potential new alternative to treat infections caused by pathogenic bacteria, particularly those caused by antibiotic-resistant bacteria. In the present study, phage YC#06 against highly multidrug-resistant Acinetobacter baumannii 4015 was isolated, identified, and characterized. Compared with antibiotics alone, the time-kill experiments *in vitro* showed that YC#06 and antibiotic mixtures that include the chloramphenicol, imipenem, and cefotaxime combination could produce phage-antibiotic synergy (PAS), which reduced the ultimate effective concentration of antibiotics. No phage-resistant bacteria have been isolated during the whole time-kill experiments *in vitro*. Of note, PAS was dose dependent, requiring a moderate phage dose to achieve maximum PAS effect. In addition, PAS could effectively inhibit biofilm formation and remove mature biofilms *in vitro*. Furthermore, PAS between the combination of YC#06 and antibiotic mixtures *in vivo* was validated using a zebrafish infection model. Overall, the results of this study demonstrate that PAC could be a viable strategy to treat infection caused by high-level multidrug-resistant Acinetobacter baumannii or other drug-resistant bacteria through switching to other types of phage and antibiotic mixtures.

**IMPORTANCE** The treatment of multidrug-resistant bacterial infection is an urgent clinical problem. The combination of bacteriophages and antibiotics could produce synergistic bactericidal effects, which could reduce the emergence of antibiotic resistance and antibiotic consumption in antibiotic-sensitive bacteria, restore efficacy to antibiotics in antibiotic-resistant bacteria, and prevent the occurrence of phage-resistant bacteria. Phage-antibiotic combination (PAC) might be a potential new alternative for clinical treatment of multidrug-resistant bacterial infections.

## INTRODUCTION

Due to the prevalence of infectious disease caused by multidrug-resistant Acinetobacter baumannii, few antibiotics are effective for treating infections caused by these bacteria. Novel and improved therapeutics against Acinetobacter baumannii infection and other multidrug-resistant bacterial infections are needed, of which phage-antibiotic combination treatments might be one promising approach (1, 2).

A bacteriophage is a virus that infects specific host bacteria. Because of their affinity and rapid proliferation characteristics, phages are widely used in the identification of pathogens (3, 4). Bacteriophages were important applications in anti-infection in the initial stage (5). With the advent of antibiotics, the role of bacteriophages in anti-infective treatment had gradually been replaced. At present, with increasing numbers of multidrug-resistant bacteria, the value of bacteriophage therapy as a potential adjunct to conventional antibiotics has been rerecognized (6, 7).

Unfortunately, bacteria also develop resistance to phages during phage therapy. Current studies indicate that combined use of bacteriophages and antibiotics could effectively control and kill bacteria and reduce the development and spread of resistant bacteria because of the phage-antibiotic synergy (PAS), which describes the phenomenon whereby sublethal concentrations of certain antibiotics can substantially stimulate the host bacteria’s production of virulent phage (8, 9). The synergistic effect of phage and antibiotic combinations has been previously described (10). However, the effects originated from both antibiotic categories and phage dose on PAS deserve attention.

The goal of this study is to develop an effective biocontrol strategy that might be used as an alternative for multidrug-resistant Acinetobacter baumannii infection. To achieve the purpose, a virulent phage (YC#06) against a highly resistant isolate of Acinetobacter baumannii 4015 (*B.m*#4015) was isolated and characterized. Subsequently, the effects of phage dose on PAS and PAS on biofilm formation and mature biofilm removal were studied. The PAS between the combination of YC#06 and antibiotic mixtures *in vivo* was also validated using a zebrafish infection model.

## RESULTS

### Susceptibility results of host bacteria *B.m*#4015.

According to the CLSI guideline (11), the antibiotic profiling of *B.m*#4015 was carried out by the standard broth microdilution method. The results showed that *B.m*#4015 was resistant to multiple antibiotics such as ciprofloxacin, gentamicin, chloramphenicol, minocycline, amikacin, imipenem, aztreonam, and cefotaxime (Table 1). The antibiotic profiles of the other isolates are shown in Table S1 in the supplemental material.

**TABLE 1:** Antibiotic resistance profiling of *B.m*#4015

Antimicrobial	Effective antibiotic concn (×MIC)^*a*^^,^^*c*^	MIC (μg/mL)	R or S^*b*^
Ciprofloxacin	>16	1	R
Gentamicin	>16	4	R
Chloramphenicol	32	8	R
Minocycline	32	4	R
Amikacin	>16	16	R
Imipenem	>16	4	R
Aztreonam	>16	8	R
Cefotaxime	>512	8	R

a Initial effective antimicrobial concentration was determined based on the microbroth dilution method.

b S, susceptible; R, resistant.

c “×MIC” shows that the effective antibiotic concentrations used are at *n* times the MIC. MICs were chosen according to the Clinical and Laboratory Standards Institute.

### Characterization and genome analysis of YC#06.

**FIG 1:**
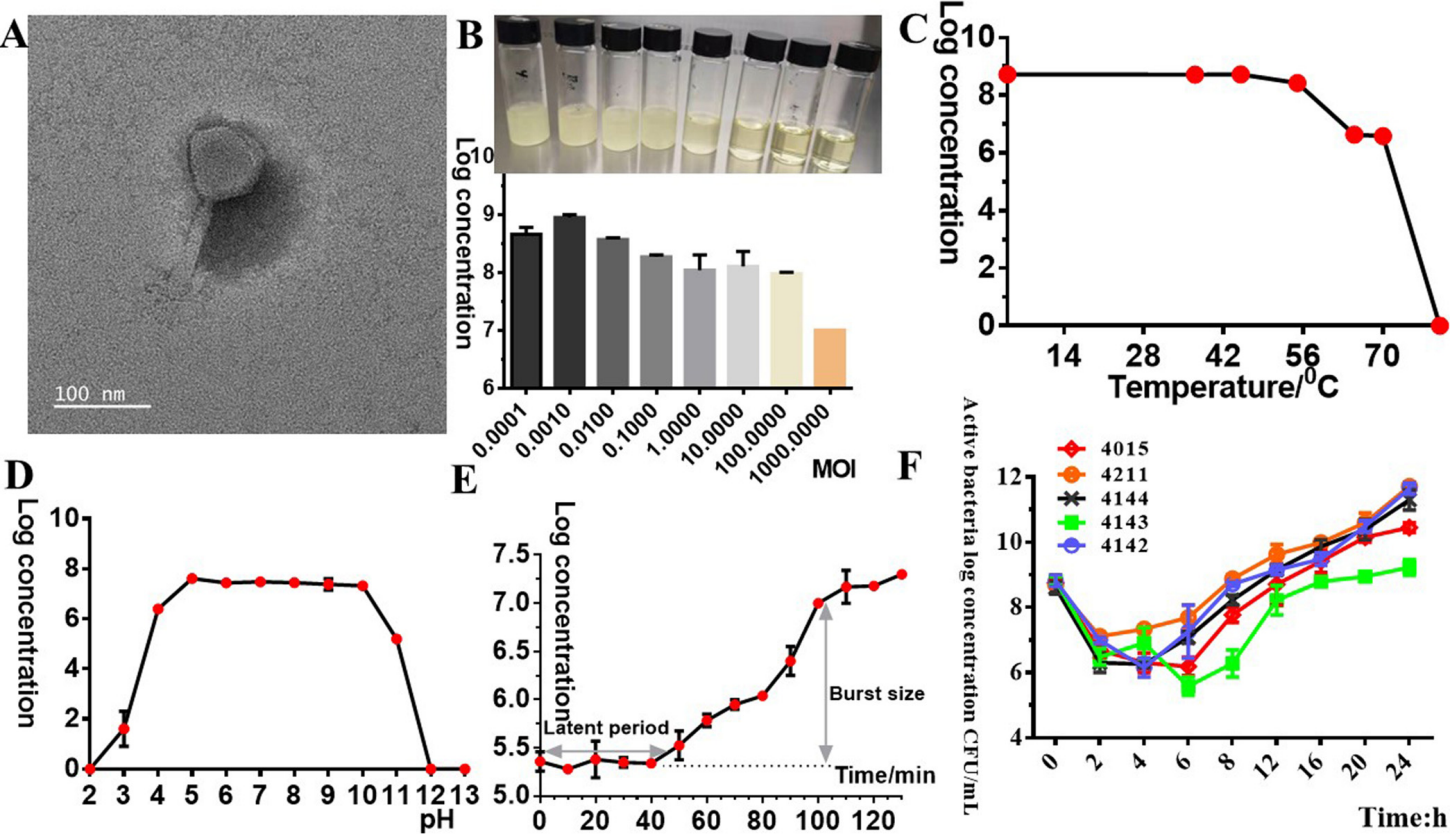
(A) Transmission electron micrograph of phage YC#06 virions negatively stained with 2% phosphotungstic acid. (B to E) Biological properties of phage YC#06. Determination of the optimal MOI (B), temperature tolerance (C), pH sensitivity (D), and one-step growth curve (E). (F) Dynamic curves of bacterial escape from phage control based on viable bacteria counting observation. Phage titers were measured by the double-layer agar method. Data are presented as the mean ± standard deviation (SD).

The plaque diameter of YC06 was measured as ~2 to 3 mm and has clear centers surrounded by a thin halo zone (Fig. S1). Transmission electron microscopy (TEM) images (Fig. 1A) indicated that the head diameter of YC#06 was approximately 81.22 ± 1.89 nm in length and 73.99 ± 1.76 nm in width. The YC#06 contractile tail was 120.09 ± 0.94 nm in length and 12.56 ± 1.02 nm in width. Thus, YC#06 showed a high probability of belonging to the family *Myoviridae*. Phage YC#06 survived steadily over a temperature range of 4 to 55°C. When the phage was incubated at 65°C for 60 min, its titer rapidly decreased to a 6-log concentration (PFU/mL), and upon incubation at 80°C for 60 min, the titer drastically decreased to 0. These results indicated that the phage could tolerate a broader range of temperature environments (Fig. 1C). Phage YC#06 maintained a good activity in the pH range of 5.0 to 10, but its titers dramatically decreased to 2 log and 5 log at pH 3.0 and 11.0, respectively. When incubated at pH 2.0 and 12.0, the phage was completely inactive. Thus, the optimum pH range for phage YC#06 was found to be 5.0 to 10.0 (Fig. 1D). Based on the maximum phage titer obtained upon infection of *B.m#*4015, the optimal multiplicity of infection (MOI) of phage YC#06 was 0.001 (Fig. 1B). The one-step growth curve of phage YC#06 showed a latent period of 40 min and a burst period of 110 min (Fig. 1E), followed by a plateau period. The size of the burst, calculated as the ratio of the average number of phages released per infected host cell before (5.36E-5 CFU) and after (7.3E-7 PFU) the burst, was approximately 116 PFU/cell after 110 min (Fig. 1E). Based on the bacterial growth detected using the dilution plate counting method in the phage-host bacteria coculture, two different growth dynamics pattern groups could be observed (Fig. 1F). For A. baumannii 4143, the bacteria were lysed and never recovered within 24 h. For A. baumannii 4015, 4211, 4142, and 4144, the bacteria were lysed for an initial 8 to 12 h and recovered gradually after 12 h, which indicated that isolates develop resistance to the phage at different phage resistant rates.

The entire YC#06 genome was sequenced. After quality control, 9,766,414 reads were obtained. The final genome assembly yielded a 48,885-bp linear double-stranded DNA (dsDNA) molecule with 40.14% G+C content, with no tRNAs identified. A total of 59 open reading frames (ORFs) larger than 100 amino acids (aa) on the draft genome using ORF Finder. The genomes were related to the phage mainly involving DNA replication, DNA packaging, restriction-modification, and lysis.

The final consensus sequence showed that it was 98.00% identical to the complete genomes of A. baumannii lytic bacteriophage Bϕ-B1251 (GenBank accession no. JX403940) in a nucleotide BLAST comparison. The phylogenetic tree was constructed based on the whole-genome sequences of phage YC#06 and other phages. The results of the phylogenetic analysis showed that phage YC#06 was the most closely related to phage Bϕ-B1251, (Fig. S3). We performed drug resistance gene analyses, and no drug resistance genes were found, suggesting that YC#06 could be safely used for therapeutic applications.

To find out the host range of phage YC#06 by spot assay, a total of 20 clinical Acinetobacter baumannii strains and Escherichia coli, Pseudomonas aeruginosa, and Streptococcus pneumoniae were used, followed by plaque assay for further confirmation. YC#06 showed activity against 5 Acinetobacter baumannii isolates, whereas no activity was observed against other strains used in this study (data not shown). Results of multilocus sequence typing (MLST) indicated that these host bacteria belonged to Acinetobacter baumannii sequence type ST2 (shown in Table S4).

### The effect of phage dose on PAS between the combination of YC#06 and antibiotics.

**FIG 2:**
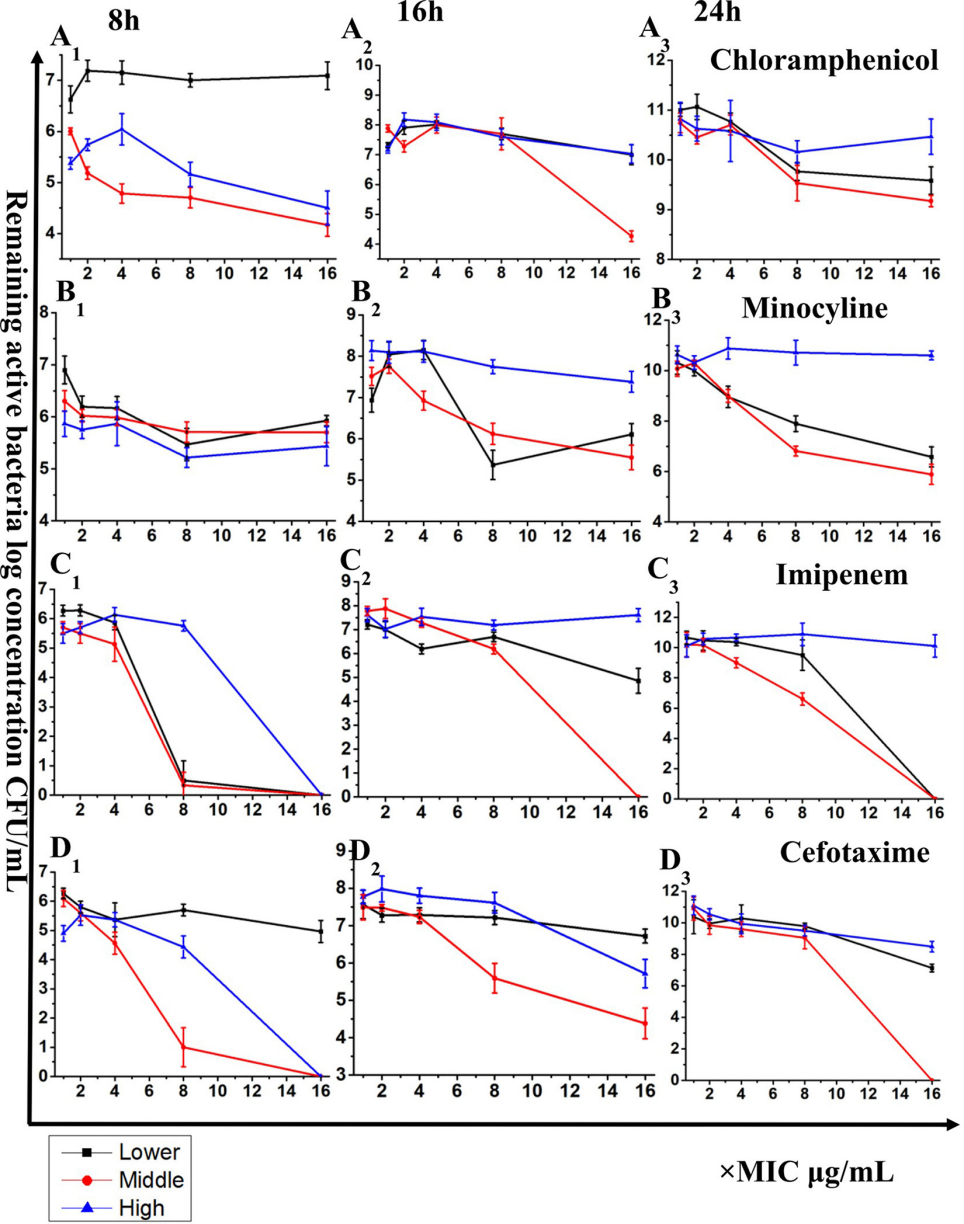
Effect of phage dose on PAS between YC#06 and antibiotic combination. The *y* axis represents the active bacteria amount, which is calculated by log (CFU/mL). The *x* axis represents the effective antibiotic concentration. “×MIC” shows that the effective antibiotic concentrations used are at *n* times the MIC. MICs were chosen according to the Clinical and Laboratory Standards Institute guidelines. Lower, phage dose at a low MOI (0.01); middle, phage dose at a middle MOI (1); high, phage dose at a high MOI (100).

To test the bactericidal effect of YC#06 combined with antibiotics, some of the drugs most commonly used against Acinetobacter baumannii infection were chosen. PAS was only found with chloramphenicol, minocycline, imipenem, and cefotaxime (Fig. S2). These data demonstrated that PAS depends on particular antibiotics. Furthermore, the bactericidal effect of chloramphenicol, minocycline, imipenem, and cefotaxime combined with different phage doses was explored. As observed in Fig. 2, the PAS of the combination of medium-dose (MOI of 1) and high-dose (MOI of 100) phage and antibiotics is obvious within the initial 8 h. The synergistic antibacterial effect of low doses (MOI of 0.01) continued to increase from 8 h to 24 h. The PAS of the combination of high-dose phage and antibiotics is further weakened with increasing incubation time. The PAS of low-dose and medium-dose phage is obvious at the 24-h time point when PAS in the medium dose is stronger. Based on these results, the phage dose, ranging from low to medium, combined with antibiotics could acquire the best synergistic antibacterial effect.

### The PAS between the combination of YC#06 and antibiotic mixture.

**FIG 3:**
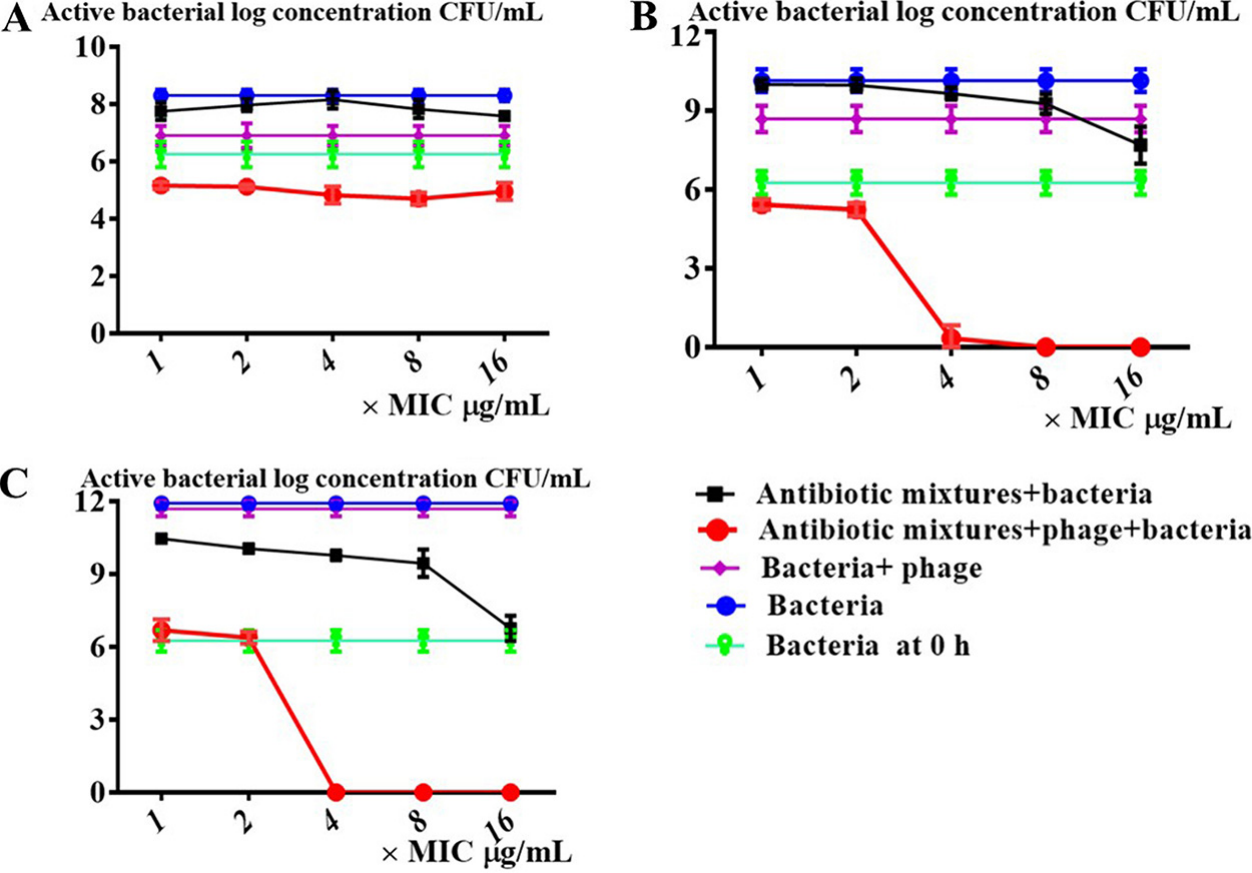
Time-kill analyses of antibiotic mixtures alone and in combination with bacteriophage YC#06 against *B.m*#4015 at different MICs at the end of 8 h exposure (A), at the end of 16 h exposure (B), and at the end of 24 h exposure (C). Antibiotic mixtures include chloramphenicol, imipenem, and cefotaxime. “×MIC” shows that the effective antibiotic concentrations used are at *n* times the MIC. MICs were chosen according to the Clinical and Laboratory Standards Institute guidelines. Synergy was defined as a 2-log_10_ CFU/mL kill compared to the most effective agent (or double-combination regimen) alone at 24 h. Bactericidal activity was defined as a 3-log_10_ CFU/mL reduction from baseline. Data are presented as the mean ± standard deviation (SD).

To confirm the possible strong synergistic activities of YC#06 with antibiotic mixtures that include chloramphenicol, minocycline, imipenem, and cefotaxime, time-killing assays were performed against the *B.m*#4015. According to the time-kill curves, although a combination of phage YC#06 and 8× MIC antibiotic mixtures from group 1 to group 11 could show bactericidal activity at 8 h and 16 h, the bactericidal activity was not observed at 24 h except for group 1, group 4, and group 5, especially in group 4. These results demonstrated that the most effective synergistic activity against log-phase bacteria involved chloramphenicol, imipenem, and cefotaxime, which are shown in Fig. S4 and Fig. 3. In YC#06 combined with 4× MIC antibiotic mixtures that include chloramphenicol, imipenem, and cefotaxime, the viable cells were not observed by the plate dilution count method at 24 h (Fig. 3C). The growth of *B.m*#4015 is totally inhibited when YC#06 is combined with 1× MIC antibiotic mixtures that include chloramphenicol, imipenem, and cefotaxime at 24 h (Fig. 3C). Further, testing if the current strategy allowed the YC#06 to kill other isolates of A. baumannii that it had previously failed to kill showed that PAS could be observed in four isolates (Table S2).

### Effect of the combination of YC#06 and antibiotic mixtures on *B.m#*4015 biofilm formation and mature biofilm reduction.

**FIG 4:**
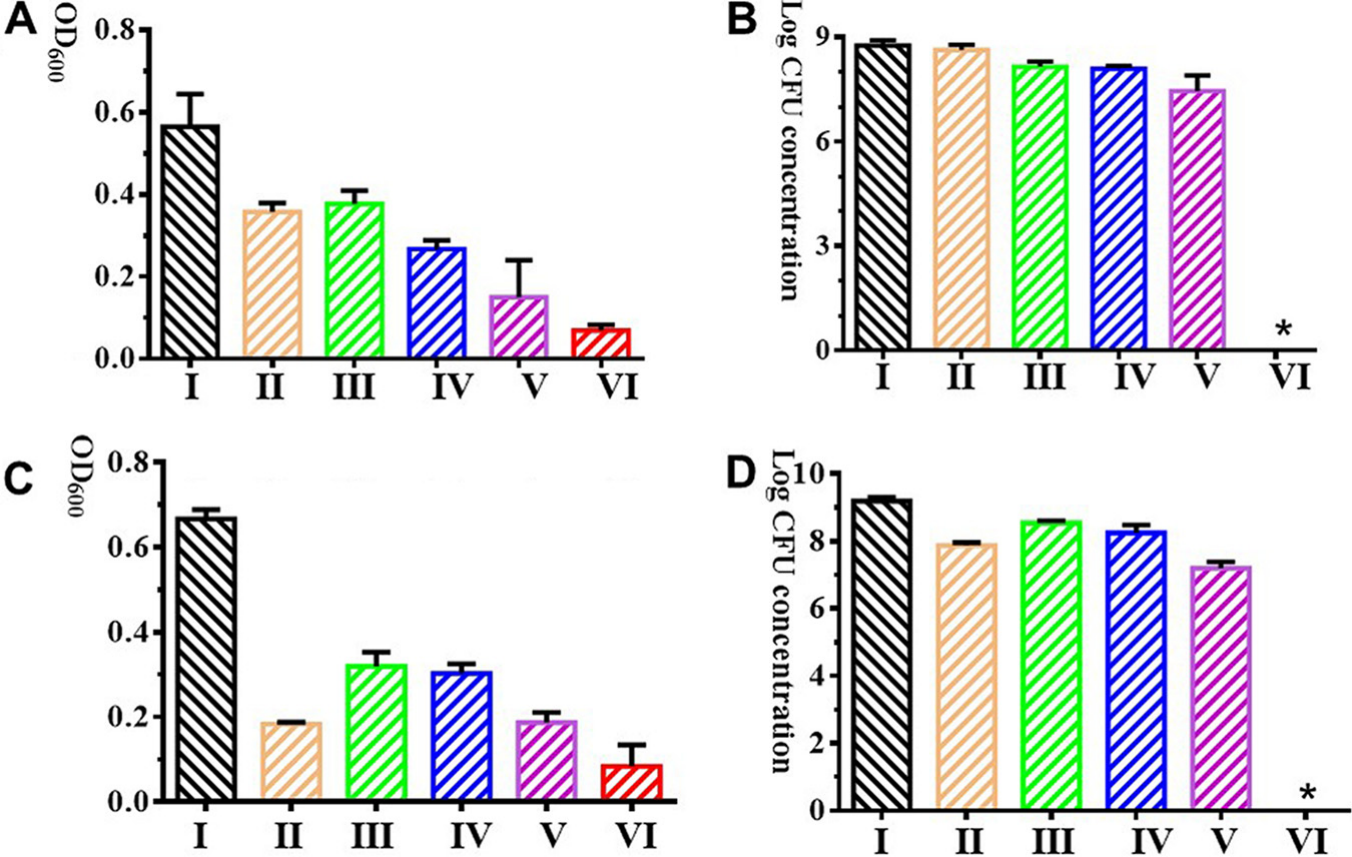
Antibiofilm effect of antibiotic mixtures alone and in combination with bacteriophage YC#06 on biofilm formation inhibition (A, B) and mature biofilm reduction (C, D) *in vitro*. Antibiotic mixtures include chloramphenicol, imipenem, and cefotaxime. (A and C) Optical density at 600 nm (OD_600_) was measured using the microplate reader. (B and D) Viable bacteria counting was detected using dilution plate counting method. I, bacteria added alone as control group; II, phage added as treatment group; III and IV, antibiotic mixtures added as treatment group; V and VI, antibiotic mixtures plus phage added as treatment group. For the biofilm formation inhibition experiment in panels A and B, the phage and *B.m*#4015, antibiotic mixtures and *B.m*#4015, and antibiotic mixtures plus phage and *B.m*#4015 were cocultured at the initial time. For the mature biofilm-reducing experiment in panels C and D, phage, antibiotic mixtures, and antibiotic mixtures plus phage were added after mature biofilm was observed. “×MIC” shows that the effective antibiotic concentrations used are at *n* times the MIC. MICs were chosen according to the Clinical and Laboratory Standards Institute guidelines. Data are presented as the mean ± standard deviation (SD) (*, *P* < 0.05).

The effect of the combination of phage YC#06 and antibiotic mixture on biofilm formation and mature biofilm reduction was assessed by crystal violet assay and the viable bacterial count method. As shown in Fig. 4A and C, phage combined with antibiotic mixtures that include chloramphenicol, imipenem, and cefotaxime (4× MIC) could inhibit biofilm formation and eliminate biofilm thoroughly. The phage combined with antibiotic mixtures (1× MIC) could inhibit the biofilm formation and eliminate the biofilm to some degree. As shown in Fig. 4B and D, when YC#06 is combined with 4× MIC antibiotic mixtures, the viable cells were not observed by the dilution plate counting method, and the growth of *B.m*#4015 was inhibited when YC#06 was combined with 1× MIC antibiotic mixtures.

### The activity of YC#06 and antibiotic mixtures alone or in combination against *B.m#*4015 in the zebrafish infection model.

**FIG 5:**
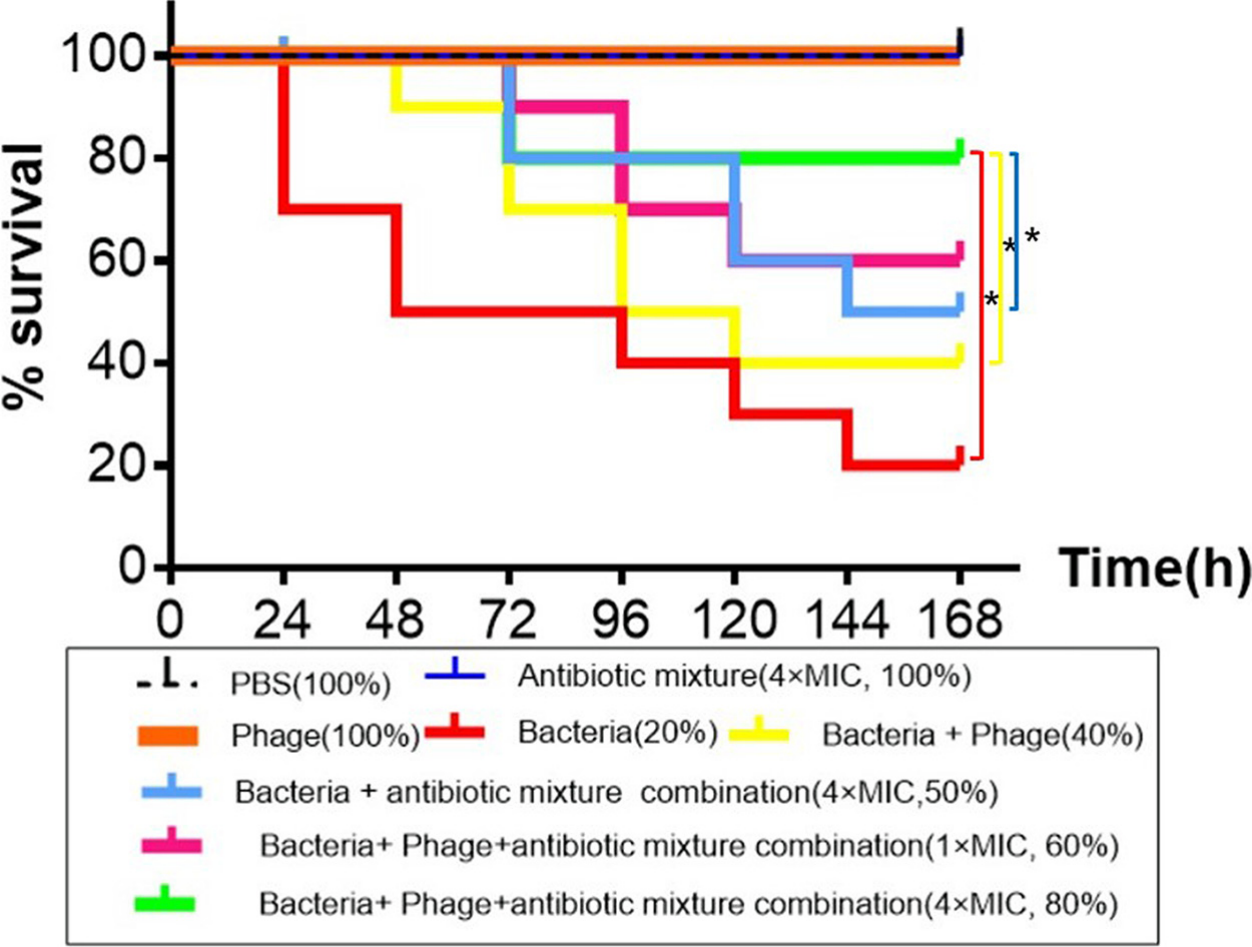
*In vivo* efficacy of YC#06 and antibiotic mixtures alone or combination against *B.m#*4015 in a zebrafish model. Monitoring lasted for a period of 7 days. Statistical analyses were performed using a *t* test for two comparisons and analysis of variance (ANOVA) test (*, *P* < 0.05).

To test whether the combination of YC#06 and antibiotic mixtures that include chloramphenicol, imipenem, and cefotaxime could prevent death from Acinetobacter baumannii infection *in vivo*, a zebrafish infection model was used. YC#06 and antibiotic mixtures that include chloramphenicol, imipenem, and cefotaxime alone against *B.m#*4015 were also monitored. Results (Fig. 5) showed that the combination of YC#06 with antibiotic mixtures (4× MIC) significantly improved (*P < *0.05) the survival rate (80%) compared with the experimental groups treated via the intraperitoneal (i.p.) route with phage alone or antibiotic alone (40% and 50%, respectively). The combination of YC#06 with antibiotic mixtures (1× MIC) also improved the survival rate (60%). Control groups, which were injected with 1× phosphate-buffered saline (PBS) at the same volume of bacterial suspension, showed 100% survival rate throughout the experimental period.

## DISCUSSION

Exploring phages and antibiotics in combined use could be useful because they may act synergistically, which could reduce the emergence of bacterial resistance and would be an effective way to improve the bactericidal activity of individual drugs. In the present study, the phage YC#06 was screened and identified based on the high-level multidrug-resistant *B.m#*4015 as the host bacteria. The whole-genome sequence alignment showed that YC#06 was 98% similar to the complete genomes of A. baumannii lytic bacteriophage Bϕ-B1251 (GenBank accession no. JX403940) (12). The G+C content (40.14%) of YC#06 is different from Bϕ-B1251 (39.05%). Compared with microorganisms commonly found on earth (temperatures between 10°C and 40°C, near-neutral pH) (13), basic characteristics of YC#06 show that the phage has strong high temperature resistance and a relatively wide pH range, which shows thatYC#06 would facilitate the development of an alternative tool to control the spread of multidrug-resistant A. baumannii. Also, the candidates of phage for biocontrol or phage therapy should be obligately lytic to bacterium and should not have integrases, where the genome of phage integrates into host DNA and coexists in it; some antibiotic-resistant genes or other host fragments would be assembled into the new synthesis phages (14, 15). The phage YC#06 is lytic, and an integrase gene is not found in the phage genome based on the functional analysis of predicted genes.

Phage therapy alone eventually leads to the emergence of phage-resistant bacteria. The results of PAS screening of 8 antibiotics revealed that the phage YC#06 combined with 4 antibiotics could produce a synergistic effect against *B.m*#4015. It is especially worth noting that YC#06 and antibiotic mixtures that include chloramphenicol, imipenem, and cefotaxime in combination could reduce the concentration of effective antibiotics (shown in Fig. 3). A related effect was also illustrated in diverse host-phage systems, including T4-like phages, with β-lactam and quinolone antibiotics, as well as mitomycin C (16, 17). The mechanism of PAS response between the combination of phage and a single antibiotic has been narrated. Significantly, the chemically unrelated quinolone antibiotic, nalidixic acid, which stimulates phage growth at levels similar to the β-lactams, also acts by inhibiting bacterial cell division (17). The bactericidal mechanism of 3 antibiotics in this study shows that 3 antibiotics target the cell wall. The mechanism of the PAS response between the combination of phage and three-antibiotic mixture still is unclear. The interpretation of antibiotic selection and PAS mechanism will help to provide better guidance for the clinical application of bacteriophages and antibiotics’ combined use in multidrug-resistant bacterial infection therapy. Otherwise, this strategy also helps phages to kill some bacteria that the phage could not kill before. For some sensitive host bacteria, it can further reduce the effective antibiotic concentration.

The dose of phage in PAC is also a factor that needs attention. Our results show that both low-dose and medium-dose phages (MOIs ranging from 0.01 to 1) can produce significant synergistic antibacterial effects. High-dose phages can also produce good synergistic effects in a short period of time, but this synergy decreases with time. The reason may be that high-dose bacteriophages have brought about a more difficult living environment for pathogens, leading to the development of bacterial resistance.

Biofilm formation is a complex process involving the initial adherence of bacterial cells to a surface, followed by the production of an extracellular matrix. Importantly, biofilm-embedded cells are known for their increased ability to withstand antibiotics and disinfectants compared to planktonic cells (18). In the current research, the combination of phage YC#06 and antibiotic mixtures that include chloramphenicol, imipenem, and cefotaxime combination cannot only effectively inhibit the formation of biofilm but also can clearly mature biofilm. The results provide a new idea for the treatment of pathogen infections that are prone to biofilm formation.

Confirmation of *in vitro* results in an animal model of infection is important for assessing the validity of the therapeutic efficacy since it is not always the synergism found *in vivo*. The zebrafish infection model further illustrates that this strategy of bacteriophages and antibiotic mixtures in combined use also has a good bactericidal effect *in vivo*, providing support for subsequent experiments *in vivo* and clinical treatment. A case of a phage-antibiotic combination that cured a 13-year-old girl who developed chronic polymicrobial biofilm infection of a pelvic bone allograft after Ewing’s sarcoma resection surgery has been reported (19). In the United States, a Food and Drug Administration (FDA)-approved phase I clinical trial was conducted (20). In 2020, researchers from the Shanghai Bacteriophage Research Institute successfully treated patients with recurrent urinary tract infection, which was caused by multidrug-resistant Klebsiella pneumoniae, by combined utilization of inactive antibiotics and bacteriophages (21). These results have further proven the potential clinical application value of this strategy in this study. Nevertheless, the immune regulation based on YC#06 and antibiotics in combination *in vivo* is still unclear and is worthy of further study.

In conclusion, in the current paper, we have demonstrated the effective antibacterial and bactericidal effects against a multidrug-resistant Acinetobacter baumannii isolate *in vitro* and *in vivo* based on PAS between the combination of phage YC#06 and antibiotics, and we provided a strategy to treat infections caused by multiresistant Acinetobacter baumannii or other drug-resistant bacteria in the clinic.

## MATERIALS AND METHODS

### Bacterial strains, culture conditions, and drug susceptibility testing.

*B.m*#4015 as the host bacteria, another 19 Acinetobacter baumannii isolates, Escherichia coli, Pseudomonas aeruginosa, and Streptococcus pneumoniae were used in this study and were isolated from the Yichang Central People’s Hospital (Hubei Province, China). These strains were grown and maintained in Luria-Bertani (LB; 10 g of NaCl, 5 g of yeast extract, 10 g of tryptone, and 15 g of agar per L, where yeast extract, tryptone, and agar were purchased from Oxoid Ltd., Basingstoke, Hampshire, England) plates at 37°C. Antimicrobial profiling of *B.m*#4015 and other isolates was performed by using the broth microdilution method (11).

### Bacteriophage isolation, purification, propagation, and host range.

A bacteriophage specific to *B.m*#4015 was isolated directly from a sewage sample collected in Yichang, Hubei Province, China. The phage was isolated using the phage enrichment technique. Briefly, double-LB broth containing 1 mM CaCl_2_, 1 mL of the log-phase host bacteria, and 100 mL of the filtered water samples was added to a 250-mL triangular flask and incubated at 37°C for 24 to 48 h. After incubation, the contents were centrifuged, and the supernatant was filtered through a 0.22-μm membrane filter (MilliporeSigma, Bedford, MA). The presence of phage was confirmed using a double-agar overlay method (3). Then, a single plaque was picked, resuspended in SM buffer (50 mM Tris-HCl, pH 7.5, 150 mM NaCl, 10 mM MgCl_2_, and 2 mM CaCl_2_), vortexed, and centrifuged. The supernatant was serially diluted and incubated with host bacteria. The phage was purified as described above. The process was repeated thrice, ensuring that plaque morphology remained the same during the iterative process. The phage isolated against *B.m*#4015 was designated YC#06.

A high-titer phage lysate was prepared by overnight propagation in a log-phase bacterial culture with shaking (180 rpm) at 37°C. The phage lysate was subsequently centrifuged at 12,000 × *g* for 10 min at 4°C, and the supernatant was filtered through a 0.22-μm membrane filter. The phage lysate was preserved at 4°C for further experiments. The phage titer was determined by means of a double-agar overlay assay as previously described (3).

A total of 20 clinical Acinetobacter baumannii isolates, including *B.m*#4015, and Escherichia coli, Pseudomonas aeruginosa, and Streptococcus pneumoniae were used to assess the host range of the isolated bacteriophage. The host range of the YC#06 was determined using a spot assay (22). Overnight *B.m*#4015 cultures were prepared in LB medium. One hundred microliters of culture were inoculated into 5 mL of molten LB top agar and overlaid onto LB agar plates. Each overlay was allowed to solidify for 15 min. Five microliters of the phage cultures of 10^8^ PFU/mL were dropped onto the overlaid top agar. After 12 h of culturing at 37°C, the presence or absence of a lysis zone was observed. Spot tests were repeated in triplicate.

### Bacteriophage characterization.

### (i) Transmission electron microscopy.

Five microliters of high-titer (10^9^ PFU/mL) phage suspension were deposited on a carbon-coated copper grid and were allowed to adsorb for 1 min. Phage particles were stained with 2% phosphotungstic acid (Beijing Solarbio Science & Technology Co., Ltd.). The carbon-coated copper grid was examined by transmission electron microscopy (TEM; JEM-2100F; Jeol Co., Tokyo, Japan).

### (ii) pH and thermal stability assays.

For pH stability tests, 100 μL of phage solution (about 7 log concentration [PFU/mL]) was mixed with 900 μL of physiological saline (pH adjusted with NaOH or HCl, ranging from 2 to 13). These mixtures were incubated at 37°C for 2 h, and the phage survival rate was determined using the double-layer agar method. For thermal stability tests, 2 mL of phage solution (about 8 log concentration [PFU/mL]) was incubated at 4°C, 37°C, 45°C, 55°C, 65°C, 70°C, and 80°C for 60 min. The phage survival rate was determined using the double-layer agar method. All tests were performed in triplicate, and the error bar represents standard error of the mean.

### (iii) Multiplicity of infection.

To determine the optimal MOI, 100 μL of phage solution (5.80 × 10^5^ PFU/mL) and 100 μL of a serially diluted *B.m*#4015 host culture (10^2^, 10^3^, 10^4^, 10^5^, 10^6^, 10^7^, 10^8^, and 10^9^ CFU/mL) were mixed with 5 mL of LB liquid medium. After incubation for 3 h at 37°C, the cells were pelleted by centrifugation at 12,000 × *g* for 10 min at 4°C, and the supernatant was filtered by a 0.22-μm membrane filter. The phage titers were measured by the double-layer agar method. The phage/bacterium ratio at which the highest phage titer could be obtained was considered the optimal MOI.

### (iv) One-step growth curve.

A one-step growth curve of the phage was performed as previously described (23). The host bacteria were harvested at the mid-exponential growth phase, centrifuged, and resuspended in SM buffer. The host bacteria were infected with YC#06 at MOI of 0.001 (phage/bacterium ratio, 10^5^ PFU/mL to 10^8^ CFU/mL) and were allowed to adsorb for 5 to 10 min at 37°C. For the removal of unabsorbed bacteriophages, the mixture was centrifuged at 12,000 × *g* for 30 s. Then, the pellet was added to LB media (5 mL) and incubated at 37°C. Two-hundred-microliter samples were taken out at 10-min intervals during incubation and were immediately diluted to determine the phage titer by using the double-layer agar method. The experiment was done in triplicate, and the error bar represents standard error of the mean.

### (v) Dynamic curves of bacterial escape from phage control based on phenotypic resistance observation.

In order to compare whether any of the isolates developed resistance to the phage YC#06, five host bacteria, including 4015, 4211, 4142, 4143, and 4144, were cocultured with YC#06 (MOI of 0.001) in LB medium (5 mL), respectively. Two-hundred-microliter samples at 0 h, 2 h, 4 h, 6 h, 8 h, 12 h, 16 h, 20 h, and 24 h were taken out for bacterial dilution plate counting observation, respectively.

### (vi) Phage DNA extraction and sequencing.

Fresh phage solution was centrifuged at 12,000 × *g* for 10 min at 4°C, and the supernatant was filtered by a 0.22-μm membrane filter. DNase I and RNase A were added to the above-described filtrated phage solution in order to remove free nucleic acids and viral proteins. Then, phage DNA was extracted using the TIANamp virus DNA/RNA kit (Tiangen Biotech [Beijing] Co., Ltd.) based on the manufacturer’s instructions. Phage genome sequencing was performed by Sangon Biotech (Shanghai) Co., Ltd., China. Genomes were sequenced with Illumina HiSeq. In total, 2 × 9,159,513 reads were generated, and raw reads were trimmed to eliminate low-quality reads and adaptor sequences using Trimmomatic (version 0.36). Clean reads were used for *de novo* assembly with SPAdes (version 3.5.0). Genome annotation using Prokka (version 1.1.0) was carried out by the sequencing service provider. Open reading frames (ORFs; minimum size of 100 amino acids) and tRNA-encoding genes were identified on phage genomes using ORF Finder. In addition, phage genomic sequences were checked for genes coding antibiotic resistance at the website https://card.mcmaster.ca/. The alignment between whole YC#06 genome sequences and other sequences downloaded from the NCBI GenBank database was generated by Geneious alignment using global alignment with free end gaps in Geneious Prime. The phylogenetic tree was constructed from the multiple-genome sequence alignment using the neighbor-joining method, also in Geneious Prime.

### Effect of phage dose on PAS.

Initially, 8 antibiotics, including ciprofloxacin, gentamicin, chloramphenicol, minocycline, amikacin, imipenem, aztreonam, and cefotaxime, alone or in combination with YC#06 were screened based on the time-killing assay *in vitro*, of which 4 antibiotics showed a certain degree of synergy with phage YC#06 (see Fig. S2 in the supplemental material). On this basis, we further explored the effect of phage dose on PAS. *B.m*#4015 (about 6 × 10^5^ CFU/mL) infected with YC#06 (MOIs ranging from 0.01, 1 to 100) and antibiotics, including chloramphenicol, minocycline, imipenem, and cefotaxime, alone or in combination were cocultured. The concentration of antibiotics used in this study ranged from 1× MIC to 16× MIC. Two-hundred-microliter samples were taken out at 0 h, 8 h, 16 h, and 24 h during incubation and were immediately diluted to determine viable bacteria using the agar dilution plate counting method. The experiment was done in triplicate, and the error bar represents standard error of the mean.

### Time-kill curve analysis for assessing the PAS between the combination of YC#06 and antibiotic mixtures.

The effectiveness of the combination of YC#06 and antibiotics was evaluated according to the time-kill curve method. Changes in bacterial count were monitored in parallel in test tubes containing the following: only bacteria (approximately 6 log concentration [CFU/mL]); bacteria and antibiotics (ranging from 1× MIC to 16× MIC); bacteria and YC#06; and bacteria, YC#06, and antibiotics (ranging from 1× MIC to 16× MIC). These test tubes were incubated at 37°C for 24 h, and bacterial counts were determined after 0 h, 8 h, 16 h, and 24 h of incubation by the agar dilution plate counting method. These plates were incubated at 37°C overnight, and bacterial colonies were counted. Results from the experiments, carried out in triplicate, were averaged and expressed as logarithms with corresponding standard errors (mean SE). Synergy was defined as a 2-log_10_ CFU/mL kill compared to the most effective agent (or double-combination regimen) alone at 24 h. Bactericidal activity was defined as a 3-log_10_ CFU/mL reduction from baseline (24).

### Evaluation of the combination of YC#06 and antibiotic mixtures on *B.m#*4015 biofilm formation inhibition and mature biofilm-reducing activity.

Antibiofilm activity of the combination of phage YC#06 and antibiotic mixtures was assessed as previously described (25). Briefly, overnight cultures of *B.m#*4015 were diluted 1:100 (vol/vol) in fresh tryptic soy broth (TSB) medium (Beijing Solarbio Science & Technology Co., Ltd.). Then, 400 μL of bacterial suspension alone, 400 μL of bacterial suspension treated with antibiotic mixtures that included chloramphenicol, imipenem, and cefotaxime (4× MIC or 1× MIC) alone, and 400 μL of bacterial suspension cultured with phage (about 6 log concentration [PFU/mL]) alone or in combination with antibiotic mixture (4× MIC or 1× MIC) were inoculated into 24-well polystyrene microtiter plates (Bkmam), and the plates were incubated for 24 h at 37°C. Total biomass was quantified by performing the crystal violet staining assay as described previously (25). Briefly, after washing the biofilm with PBS, 1 mL of 1% (wt/vol) crystal violet was added to each well. Five minutes later, the excess crystal violet was removed by washing twice with water. The remaining dye was then solubilized by adding glacial acetic acid (33%; Merck) and ethanol (2:8), and the absorbance at 600 nm was measured using a Tecan Sunrise microplate reader.

The effect of the combination of phage YC#06 and antibiotic mixtures on mature biofilm was also studied. Overnight cultures of *B.m#*4015 were diluted 1:100 (vol/vol) in fresh TSB medium. Then, 400 μL of bacterial suspension was inoculated into 24-well polystyrene microtiter plates (Bkmam), and the plates were incubated for 24 h at 37°C. Afterward, the planktonic phase was removed, and the biofilms were washed twice with phosphate-buffered saline (PBS; 137 mM NaCl, 2.7 mM KCl, 10 mM Na2HPO4, and 2 mM KH2PO4 [pH 7.4]). The remaining adhered cells were then treated with 0.4 mL of TSB medium alone or using the same medium with antibiotic mixture (4× MIC or 1× MIC) alone or phage (about 1.2 × 10^8^ PFU/mL) alone or in combination with antibiotic mixture (4× MIC or 1× MIC) at 37°C. Treatment was allowed to act for 6 h. Then, the planktonic phase was removed, and the adhered phase was washed twice with PBS. Total biomass was quantified by performing the crystal violet staining assay as described above.

To assess the efficacy of the different treatments, the number of viable attached cells was also quantified. The number of viable cells present in the biofilms was determined by using the spot test. Briefly, biofilms were scraped and resuspended in PBS. Afterward, 10-μL droplets from 10-fold serial dilutions of this cell suspension were spotted onto LB agar plates and allowed to dry. These plates were then incubated at 37°C for 24 h and counted.

### Zebrafish model of *B.m#*4015 infection *in vivo*.

The combination of phage and antibiotic mixtures therapy efficacy was investigated by evaluating protection efficacy in zebrafish against *B.m#*4015. Wild-type (AB) adult zebrafish were maintained in water with 14/10-h light/dark cycles at 28 ± 0.5°C. Zebrafish (*n* = 10 per group) were infected via the intraperitoneal (i.p.) route with 10 μL of PBS, 10 μL of antibiotic mixtures (4× MIC), 10 μL of phage as control groups, and 10 μL of 5 × 10^5^ CFU/mL suspension of *B.m#*4015 as experimental groups. One hour postinfection, experimental groups of adult zebrafish infected with bacteria were divided into five treatment groups and were treated via the i.p. route with 10 μL of PBS, 10 μL of antibiotic mixture (4× MIC), or 10 μL of phage alone or in combination with antibiotic mixtures (4× MIC or 1× MIC). These control groups were also treated via the i.p. route with 10 μL of PBS. The survival rate for each group was monitored every 24 h for up to 7 days postinfection.

### Statistical analysis.

All experimental data were analyzed using GraphPad Prism software version 6.0 (GraphPad Software, Inc., USA). Differences with *P* values of <0.05 were considered significant at 95% confidence intervals.

### Ethical approval.

The study was approved by the Ethics Committees of Yichang Central People’s Hospital.

### Data availability.

The data that support the findings of this study are available on request from the corresponding author. The data are not publicly available due to privacy and ethical restrictions. The metagenomic database of YC#06 is available at the URL https://pan.baidu.com/s/1mTGOZTX2aWDdOfbwOosAnQ?pwd=61vm. The genome sequence of phageYC#06 has been deposited to the GenBank database, and the accession number is ON391949.1.
