# Supplementary material for: Bactericidal Synergism between Phage YC#06 and Antibiotics: a Combination Strategy to Target Multidrug-Resistant Acinetobacter baumannii In Vitro and In Vivo
Source: Microbiol Spectr. 2022 Jun 23;10(4):e00096-22. doi: 10.1128/spectrum.00096-22 (PMC9430793; doi:10.1128/spectrum.00096-22)
Supplement: Supplemental file 1 — Supplemental material. Download spectrum.00096-22-s0001.pdf, PDF file, 0.7 MB [file spectrum.00096-22-s0001.pdf]

Supplementary materials

Bactericidal synergism between phage YC#06 and antibiotics: a combination strategy to target multi-drug resistant *Acinetobacter baumannii* in vitro and in vivo

Jun Luo[a,b,c#+], Libo Xie[a,b+], Min Liu[a, b], Qianyuan Li[a, b], Peng Wang[a, b], Chunhua Luo[a,b#]

[a] The First College of Clinical Medical Science, China Three Gorges University, Yichang, China

[b] Yichang Central People's Hospital, China

[c] Central Laboratory, The First College of Clinical Medical Science, China Three Gorges University & Yichang Central People's Hospital, Yichang 443003, China

#To whom correspondence should be addressed.

Jun Luo Email: lj1988cby@126.com Tel/Fax: +86 0717-6483519

Chunhua Luo Email: lchlgj2004@aliyun.com Tel/Fax: +86 0717-6483519

Table S1 Antibiotic resistance profiling of total of 20 clinical *Acinetobacter baumannii* isolates

**Strain**	**Ciprofloxacin**		**Gentamicin**		**Chloramphenicol**		**Minocycline**		**Amikacin**		**Imipenem**		**Aztreonam**		**Cefotaxime**	
	×MIC	R/S	×MIC	R/S	×MIC	R/S	×MIC	R/S	×MIC	R/S	×MIC	R/S	×MIC	R/S	×MIC	R/S
**3014**	>16	R	>16	R	16	R	8	R	4	R	>16	R	>16	R	>16	R
**#0125**	>16	R	>16	R	>16	R	>16		8	R	16	R	>16	R	>16	R
**2101**	>16	R	>16	R	16	R	8	R	8	R	16	R	>16	R	>16	R
**1113**	<1	S	<1	S	1	S	<1	S	<1	S	4	R	<1	S	1	S
**1131**	>16	R	8	R	0.5	S	<1	S	<1	S	0.25	S	<1	S	1	S
**4142**	>16	R	>16	R	8	R	8	R	8	R	>16	R	>16	R	>16	R
**#0025**	>16	R	>16	R	8	R	8	R	8	R	>16	R	>16	R	>16	R
**#091**	>16	R	<1	S	1	S	<1	S	16	R	2	R	<1	S	8	R
**1130**	<1	S	16	R	8	R	<1	S	<1	S	0.5	S	<1	S	0.5	S
**1112**	<1	S	<1	S	0.25	S	<1	S	<1	S	1	S	<1	S	0.5	S
**4141**	>16	R	>16	R	16	R	8	R	8	R	>16	R	>16	R	>16	R
**4144**	>16	R	>16	R	>16	R	4	R	8	R	>16	R	>16	R	>16	R
**4143**	>16	R	>16	R	16	R	4	R	16	R	>16	R	>16	R	>16	R
**4211**	>16	R	>16	R	8	R	4	R	4	R	16	R	8	R	16	R
**3014**	>16	R	>16	R	8	R	4	R	4	R	16	R	>16	R	>16	R
**3214**	>16	R	>16	R	16	R	16	R	4	R	16	R	>16	R	>16	R
**#0508**	>16	R	>16	R	4	R	8	R	8	R	16	R	>16	R	>16	R
**#005**	>16	R	>16	R	16	R	16	R	8	R	16	R	>16	R	>16	R
**3708**	<1	S	16	R	8	R	4	R	8	R	>16	R	>16	R	>16	R
**#4015**	>16	R	>16	R	32	R	32	R	>16	R	>16	R	>16	R	>512	R

Note: 1. Antimicrobial profiling of 20 isolates was performed by the microbroth dilution method. S = susceptible and R = resistant.

2.N×MIC show that the effective antibiotic concentration used are at N-times MIC. MIC: minimum inhibitory concentration. MIC were chosen according to the Clinical and Laboratory Standards Institute.

Table S2 The testing results of the current strategy on other isolates of *A. baumanii*

Strain	Chloramphenicol		Imipenem		Cefotaxime		(Chloramphenicol+Imipenem+Cefotaxime)/×MIC	YC#06	PAS
	×MIC	R/S	×MIC	R/S	×MIC	R/S			
**3014**	16	R	>16	R	>16	R	8	/	**Y**
**#0125**	>16	R	16	R	>16	R	16	/	**N**
**2101**	16	R	16	R	>16	R	8	/	**Y**
**1113**	1	S	4	R	1	S	0.25	/	**Y**
**1131**	0.5	S	0.25	S	1	S	0.25	/	**N**
**4142**	8	R	>16	R	>16	R	2	+	**Y**
**#0025**	8	R	>16	R	>16	R	8	/	**N**
**#091**	1	S	2	R	8	R	1	/	**N**
**1130**	8	R	0.5	S	0.5	S	0.5	/	**N**
**1112**	0.25	S	1	S	0.5	S	0.25	/	**N**
**4141**	16	R	>16	R	>16	R	1	/	**Y**
**4144**	>16	R	>16	R	>16	R	1	+	**Y**
**4143**	16	R	>16	R	>16	R	1	+	**Y**
**4211**	8	R	16	R	16	R	4	+	**Y**
**3014**	8	R	16	R	>16	R	8	/	**N**
**3214**	16	R	16	R	>16	R	8	/	**N**
**#0508**	4	R	16	R	>16	R	4	/	**N**
**#005**	16	R	16	R	>16	R	16	/	**N**
**3708**	8	R	>16	R	>16	R	8	/	**N**
**#4015**	32	R	>16	R	>512	R	1	+	**Y**

Note: 1. Antimicrobial profiling of 20 isolates was performed by the microbroth dilution method. S = susceptible and R = resistant.

2.N×MIC show that the effective antibiotic concentration used are at N-times MIC. MIC: minimum inhibitory concentration. MIC were chosen according to the Clinical and Laboratory Standards Institute.

3. PAS was determined at the end of 24 h time-kill analyses based on the bacterial dilution plate counting observation (Data not shown). Synergy was defined as a 2-log10-CFU/ml kill compared to the most effective agent (or double-combination regimen) alone at 24 h. Bactericidal activity was defined as a 3-log10-CFU/ml reduction from baseline.

4. /: Not host bacteria; +: host bacteria.

MLST typing

The primers were listed in the Table S3 and synthesized by Sangon Biotech Co.,Ltd. (Shanghai, China). The PCR mixture had a final volume of 50 μL containing 0.25 μL of TaKaRa Ex Taq (product code: RR001A, Takara, Dalian, China), 5 μL of 10×Ex Taq Buffer, 2.5 μL of primers (20 μM), 40.25 μL of distilled ddH2O and 2 μL of the DNA template. Dnase-free water was used as a negative control. The PCR was run under the conditions as following: an initial denaturation step at 94 ºC for 5 min followed by 35 cycles of denaturation at 94 ºC for 30 s, annealing at 50 ºC for 30 s, extending at 72 ºC for 1 min, terminating the extension at 72 ℃ for 2 min, on a ABI VeritiPro PCR instrument. The purification and sequencing of PCR products were completed by Sangon Biotech Co.,Ltd.

The DNA sequences obtained by forward and reverse sequencing were spliced, and the relevant sequencing files were uploaded to the website: https://pubmlst.org/bigsdb?db=pubmlst_abaumannii_pasteur_seqdef&set_id=2&page=sequenceQuery, and blast comparison was carried out to determine the alleles of each sequence and the sequence types of each strain.

Table S3 Primers for seven housekeeping genes of MLST

Gene		primer 5'-3'	PCR product length
cpn60	F	ACTGTACTTGCTCAAGC	405
	R	TTCAGCGATGATAAGAAGTGG	
fusA	F	ATCGGTATTTCTGCKCACATYGAT	633
	R	CCAACATACKYTGWACACCTTTTTT	
gltA	F	AATTTACAGTGGCACATTAGGTCCC	483
	R	GCAGAGATACCAGCAGAGATACACG	
pyrG	F	GGTGTTGTTTCATCACTAGGWAAAGG	297
	R	ATAAATGGTAAAGAYTCGAT R TCACCMA	
recA	F	CCTGAATCTTCYGGTAAAAC	372
	R	GTTTCTGGGCTGCCAAACATTAC	
rplB	F	GTAGAGCGTATTGAATACGATCCTAACC	330
	R	CACCACCACC R TGYGGGTGATC	
rpoB	F	GGCGAAATGGC(AGT) GA(AG) AACCA	456
	R	GA(AG) TC(CT) TCGAAGTTGTAACC	

As shown in Table S4, all of five host bacteria in this experiment belong to ST2.

Table S4 Allele coding and sequence type of 5 *Acinetobacter baumannii* host bacteria

**Starin**	***cpn60***	***fusA***	***gltA***	***pyrG***	***recA***	***rplB***	***rpoB***	**ST**
4142	2	2	2	2	2	2	2	2
4143	2	2	2	2	2	2	2	2
4144	2	2	2	2	2	2	2	2
4211	2	2	2	2	2	2	2	2
4015	2	2	2	2	2	2	2	2

FIG S1 Soft agar overlay assay for phage isolation and confirmation

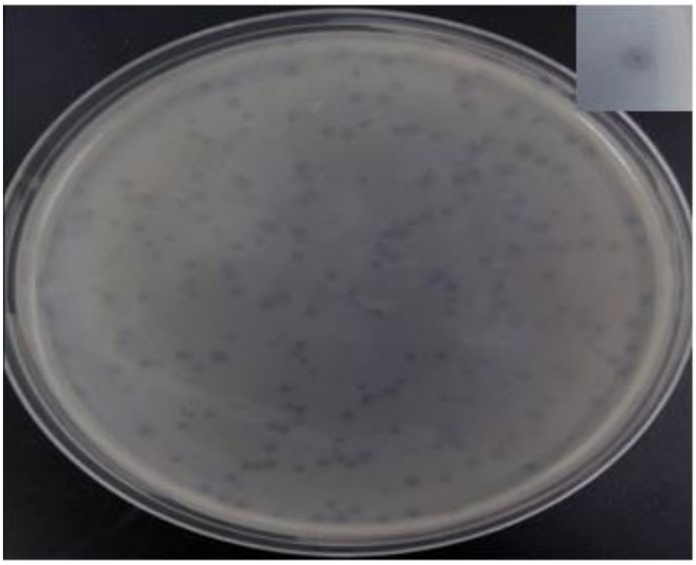

FIG S2 The efficacy of YC#06 and antibiotic mixtures alone or combination against *B.m*# 4015.

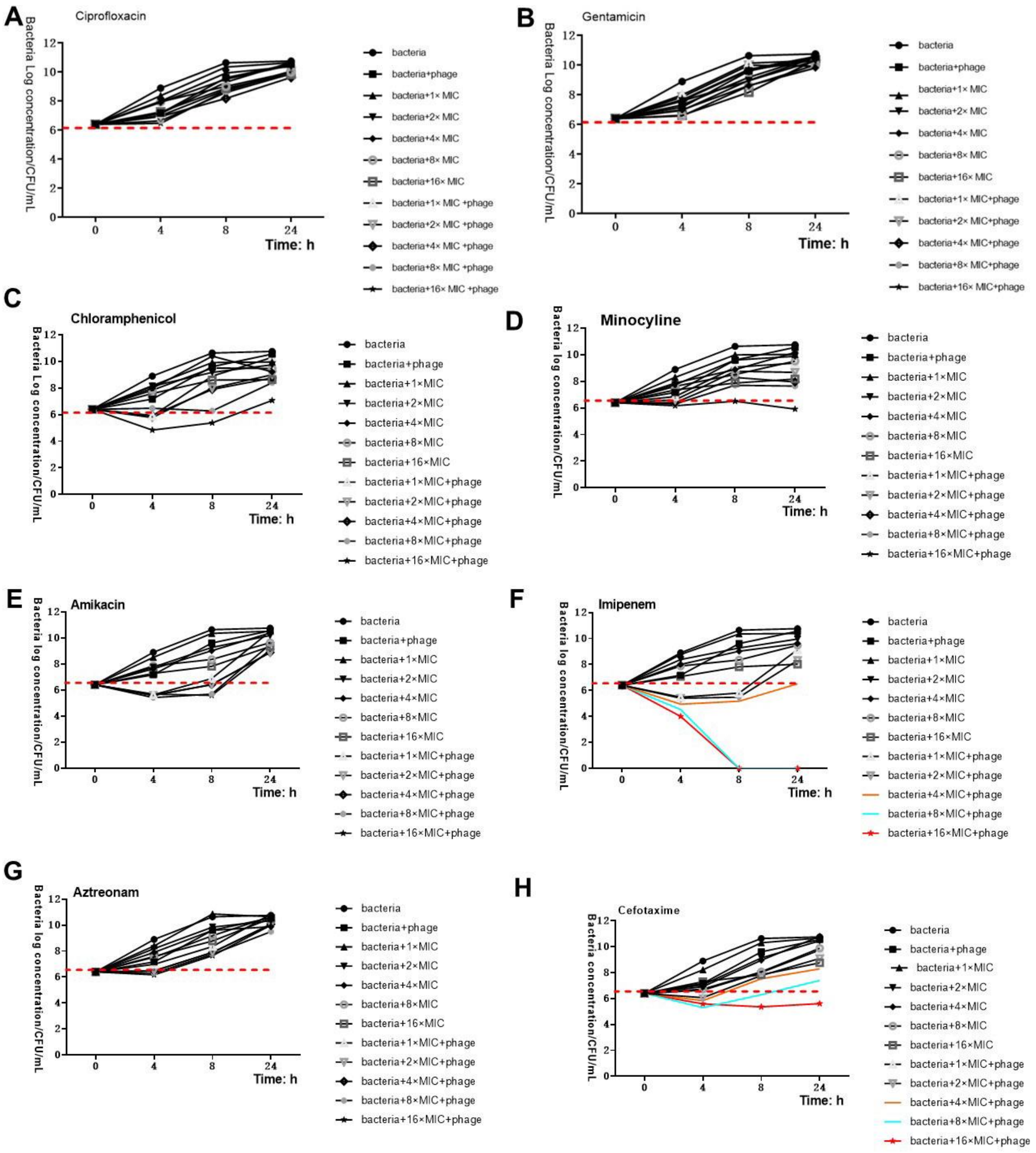

Note: ciprofloxacin, B. gentamicin, C. chloramphenicol, D. minocycline, E. amikacin, F. imipenem, G. aztreonam and H. cefotaxime

N×MIC show that the effective antibiotic concentration used are at N-times MIC. MIC: minimum inhibitory concentration. MIC were chosen according to the Clinical and Laboratory Standards Institute.

FIG S3 Phylogenetic tree

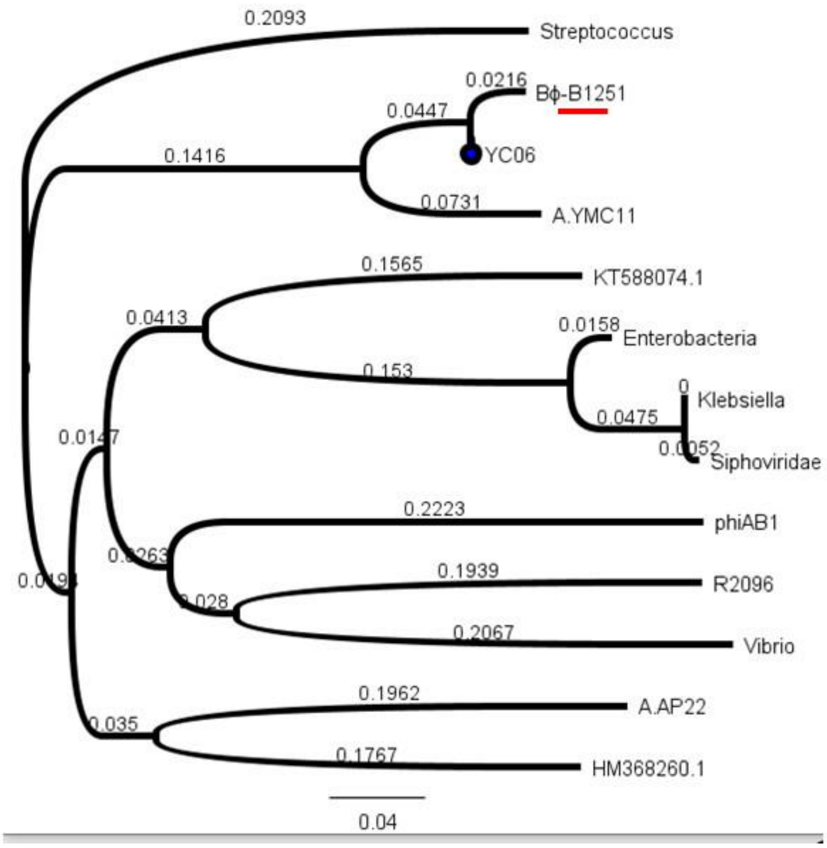

FIG S4 Time-kill curve analysis of antibiotics and bacteriophage YC#06 alone or combination against *B.m #4015*. A. observed at 8 h, B. observed at 16 h, C. observed at 24h.

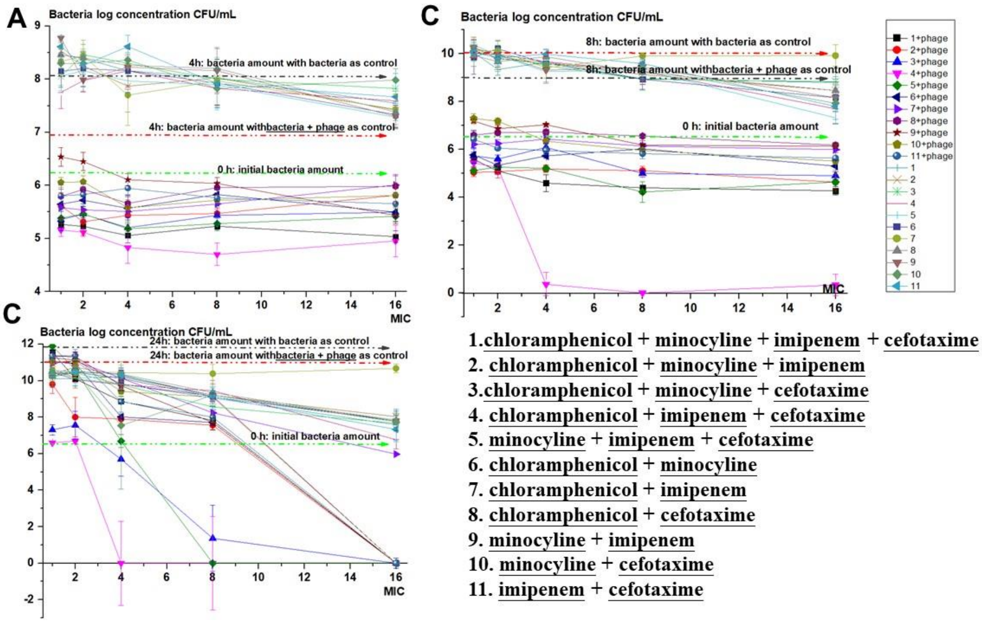

Note: N×MIC show that the effective antibiotic concentration used are at N-times MIC. MIC: minimum inhibitory concentration. MIC were chosen according to the Clinical and Laboratory Standards Institute.

Synergy was defined as a 2-log10-CFU/ml kill compared to the most effective agent (or double-combination regimen) alone at 24 h. Bactericidal activity was defined as a 3-log10-CFU/ml reduction from baseline. Values are mean plus standard deviations.
